# Individualized online exercise therapy aids recovery in pediatric long-COVID—findings from an exploratory randomized controlled trial

**DOI:** 10.1007/s00431-025-06705-5

**Published:** 2026-01-06

**Authors:** Sarah Christina Goretzki, Mara Bergelt, Laurent Weis, Rayan Hojeii, Gabriele Gauß, Miriam Götte, Ronja Beller, Sven Benson, Anne Schönecker, Adela Della Marina, Andrea Gangfuß, Florian Stehling, Christina Pentek, Anna von Loewenich, Tom Hühne, Clara Held, Sebastian Voigt, Ursula Felderhoff-Müser, Michael M. Schündeln, Nora Bruns, Katharina Eckert, Christian Dohna-Schwake, Maire Brasseler

**Affiliations:** 1 Department of Pediatrics I, Neonatology, Pediatric Intensive Care, Pediatric Infectiology, Pediatric Neurology, University Duisburg-Essen, Children’s Hospital Essen, 45147 Essen, Germany; 2 West German Centre for Infectious Diseases (WZI), University Hospital Essen, University Duisburg-Essen, Hufelandstraße 55, 45147 Essen, Germany; 3 IST University of Applied Sciences, Health Management and Public Health, Düsseldorf, Germany; 4 Department of Pediatrics III, Pediatric Hematology and Oncology, Pulmonology and Sleep Medicine, Pediatric Rheumatology and Pediatric Cardiology, University Duisburg-Essen, Children’s Hospital Essen, 45147 Essen, Germany; 5 West German Cancer Center (WTZ), University Hospital Essen, 45147 Essen, Germany; 6 Institute of Medical Psychology and Behavioral Immunobiology, University Hospital Essen, University of Duisburg-Essen, Essen, Germany; 7 Department of Pediatric Neurology, Centre for Neuromuscular Disorders, University Duisburg-Essen, Essen, Germany; 8 Institute of Virology, University Hospital Essen, University of Duisburg-Essen, 45147 Essen, Germany

**Keywords:** Pediatric long-COVID, Exercise therapy, Fatigue, Physical health, Mental health, School participation

## Abstract

The purpose of this study is to evaluate the feasibility, safety, and effectiveness of an individualized online exercise therapy (IOET) designed to improve physical capacity and quality of life in children and adolescents with long-COVID. In a prospective, randomized, single-center exploratory trial, 14 patients aged 9–17 years with long-COVID (median symptom duration: 21 months) received either 6 or 12 weeks of IOET. Sessions were held twice weekly via telemedicine and individually adapted to physical ability and symptoms. Primary outcomes were functional performance (6-minute walk test [6MWT], sit-to-stand test [STST], and handgrip strength test [HST]). Secondary outcomes included school attendance, quality of life (PedsQL), safety, and self-reported recovery. All participants showed clinically improvements. In the 12-week IOET group, 6MWT increased from 396.0 to 616.3 m (+ 220.3 m, 95% CI 98.2–342.4), STST from 25.4 to 32.6 repetitions (+ 7.2, 1.9–12.5), and HST from 16.6 to 27.1 kg (+ 10.5 kg, 4.8–16.1). The 6-week group improved comparably (6MWT: 429.0 m to 601.6 m, (+ 172.6 m, 64.7–280.6); STST: 21.6 to 31.7 (+ 10.1, 3.1–17.1); HST: 17.3 to 22.1 kg (+ 4.8 kg (0.7–8.9)). School attendance rose from 58 to 97%, and PedsQL reflected improved quality of life and reduced fatigue. No adverse events or post-exertional symptom exacerbations occurred. Improvements persisted at the 3-month follow-up. *Conclusions*: IOET is feasible, safe, and associated with improved physical function, reintegration in everyday life, and its quality in pediatric long-COVID. These findings highlight IOET as a promising rehabilitation strategy and justify larger multicenter trials to confirm effectiveness and define optimal duration.

**Table:** 

**What is Known:** • *Children and adolescents with long-COVID often experience persistent fatigue, impaired physical capacity, and reduced quality of life, with limited evidence-based treatment options available.* • *Exercise therapy has shown beneficial effects in other chronic pediatric conditions such as cancer- or fatigue-related syndromes, improving strength, well-being, and social participation.*	
**What is New:** • *This exploratory randomized controlled trial demonstrates that individualized online exercise therapy is feasible, safe, and associated with clinically relevant improvements in physical function, quality of life, and school attendance in pediatric long-COVID, without negative side effects.* • *The findings highlight the potential of telemedicine-based rehabilitation strategies as accessible and effective treatment approaches for children and adolescents with post-infectious conditions such as long-COVID. *

**Supplementary Information:**

The online version contains supplementary material available at 10.1007/s00431-025-06705-5.

## Introduction

The challenging times of the COVID-19 pandemic lasted from December 2019 to May 2023. Although children and adolescents were generally less severely affected by acute infection, post-infectious and post-viral complications have continued to pose significant challenges in pediatrics [1]. Behnood et al. (2022) showed that children may experience long-lasting symptoms, including fatigue, sleep, and emotional difficulties, months after SARS-CoV-2 infection, consistent with recent evidence [2–4].

Long-COVID, also referred to as post-COVID syndrome or post-acute sequelae of SARS-CoV-2 infection (PASC), affects children across all age groups and can substantially impair participation in everyday life, school attendance, and social interactions [5, 6]. Common symptoms include fatigue, headaches, myalgia or weakness, insomnia, concentration difficulties, and reduced quality of life and mental well-being [7, 8]. Hassan et al. reported increased mental health concerns in children with previous SARS-CoV-2 infection, including anxiety, depression, concentration and sleep disturbances, mood swings, and appetite loss [9]. As no causal treatment exists, many affected children and their families face a considerable physical and emotional burden [10].

Previous studies on pediatric chronic fatigue showed that individualized exercise therapy, adapted to the children’s and adolescents’ level of exertion, is feasible and the most effective strategy to reduce this fatigability [11]. Systematic reviews conclude that structured physical activity interventions can reduce fatigue and improve physical functioning in young people, with a generally favorable safety profile [12–14]. Exercise therapy also shows positive effects in psychosomatic illnesses [15]. Data on adult patients with long-COVID indicate that individualized physical activity can positively influence and shorten the course of the illness [16, 17]. The joy of exercise therapy is inevitable to avoid the experience of failure and to enable a long-term positive commitment [14].

The aim of this study was to evaluate the effect of individualized online exercise therapy (IOET) on physical strength and quality of life in children and adolescents with long-COVID as a single-center proof-of-concept. We conducted a randomized controlled trial assessing psychosocial factors, including depressive symptoms, and physical performance (handgrip strength (HST), 6-minute walk (6MWT), sit-to-stand (STST)) before, during, and after 6 and 12 weeks.

## Methods

### Study design and setting

**Fig. 1:**
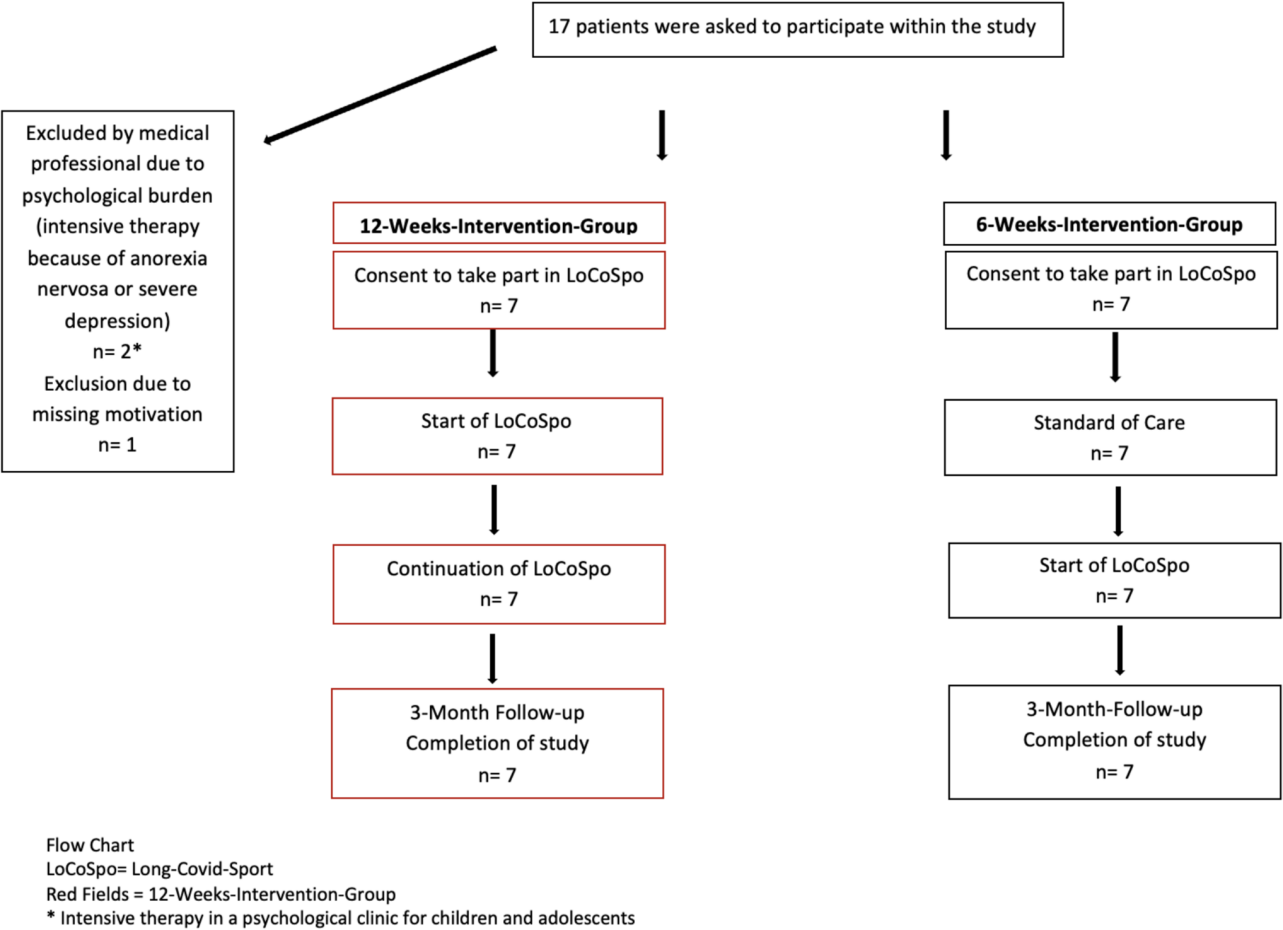
Patient enrollment and study flow

This prospective, randomized, single-center exploratory controlled trial was conducted at the University Hospital Essen, Germany. Participants were randomly assigned in a 1:1 ratio to either the intervention or control group using a computer-generated randomization system (http://www.randomized.at/) based on the minimization method. Allocation was implemented through the secure web-based platform, which generated the group assignment only after participant entry into the database, thereby ensuring allocation concealment and preventing investigators from predicting or influencing group assignment. The intervention group received a 12-week online exercise program (directly after inclusion in the study), while the second control group only received a 6-week online exercise program after a 6-week exercise-free period following recruitment as illustrated in Fig 1.

### Participants and recruitment

Participants older than 6 years, who met the long-COVID criteria, were recruited from the pediatric infectious diseases outpatient clinic from May 2023 to July 2023.

### Diagnostic work-up and baseline assessments

All participants underwent an interdisciplinary diagnostic workup (including cardiological, pulmonological, psychological, and neuropediatric assessments).

The study excluded patients with severe heart or lung function restrictions or a Bell Chronic Fatigue and Immune Dysfunction Syndrome (CFIDS) Disability Scale under 30. A total of 17 participants were approached, with 14 meeting the inclusion criteria who were included in the study.

In addition to the initial work-up, all participants received standardized cardiologic and pneumological diagnostics to exclude new dysfunctions. A summary of the standardized work-up can be found within supplement 1.

### Long-COVID terminology and classification

We decided on the term long-COVID, rather than post-COVID syndrome or post-acute sequelae of severe acute respiratory syndrome coronavirus 2 infection (PACS), as it was commonly used within the pediatric patients’ populations at the time of the study. All participants met the criteria for long-COVID as defined in Table 1, and infections were confirmed using PCR, IgG detection (including N and S) [18–20].

**Table 1:** Definitions used for long-COVID

AWMF	Persisting symptoms after acute COVID-19 or its treatment, symptoms leading to a new health impairment, new symptoms that appeared after the end of the acute phase because of a COVID-19 disease, or worsening of a pre-existing underlying condition [18]
NICE	Post COVID is described as ongoing symptoms that developed during COVID-19 and have no other explanation from 4 weeks up to 12 weeks after the acute infection, with > 12 weeks = post-COVID syndrome. It usually presents with clusters of symptoms, often overlapping, which can fluctuate and change over time and affect any system in the body [19]
NASEM	Long-COVID is an infection-associated chronic condition that occurs after SARS-CoV-2 infection and is present for at least 3 months as a continuous, relapsing and remitting, or progressive disease state that affects one or more organ systems [20]

### Outcome measures

Primary outcomes were defined as a group, with one (6MWT) designated as the lead.

Secondary outcomes included school attendance, health-related quality of life (measured using the Pediatric Quality of Life Inventory™ (PedsQL™) questionnaire), engagement in additional physical activity (daily diary recorded physical education attendance, as well as general physical activities), school attendance (daily diary recorded how many school hours were planned and how many were completed; official school absence notes were additionally collected at the end of the study), safety monitoring through laboratory parameters, and self-reported recovery from long-COVID symptoms.

### Study visits and assessment procedures

**Fig. 2:**
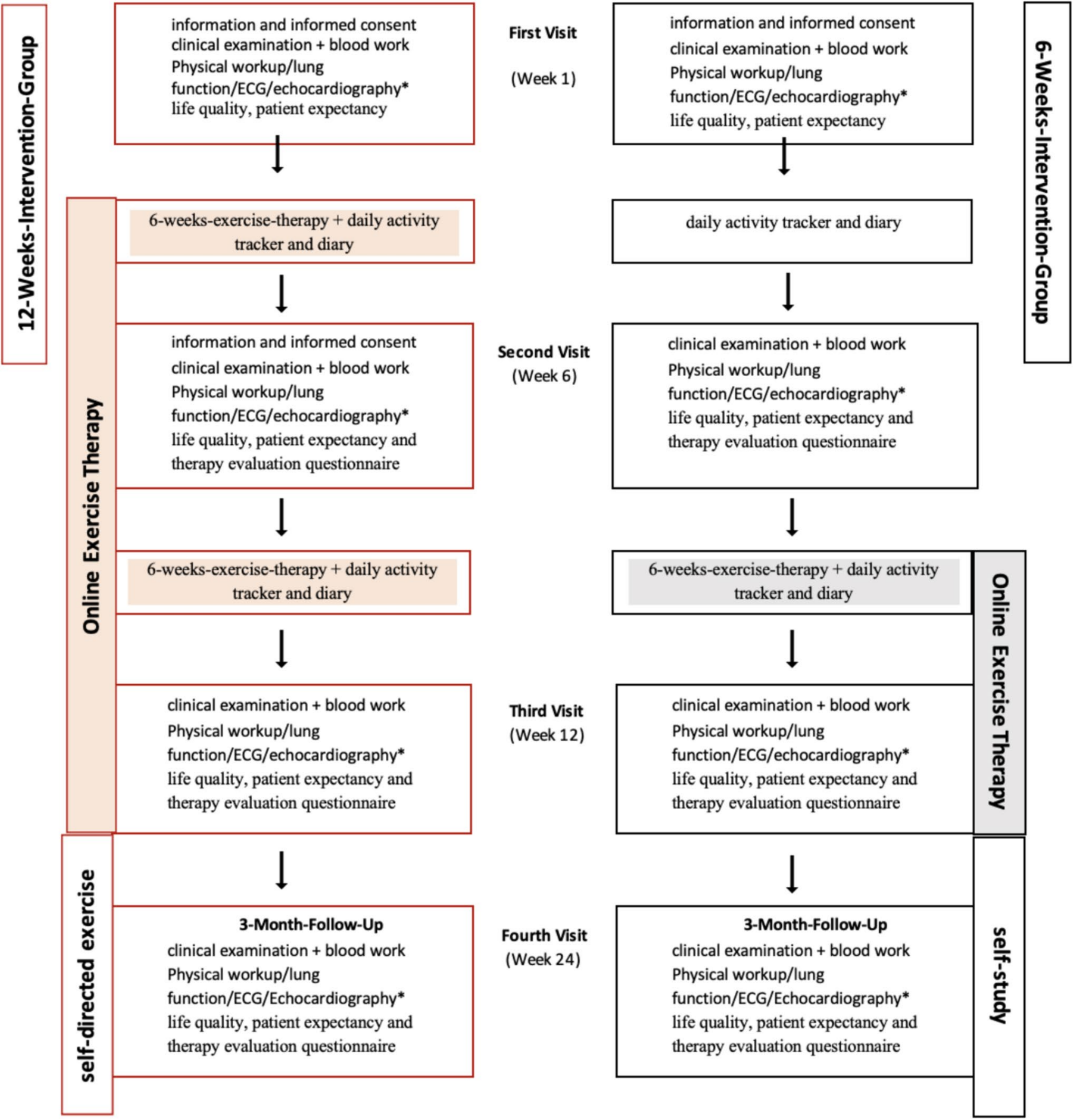
Study protocol

All participants attended four visits at the outpatient clinic throughout the study (Fig. 2). These visits were scheduled at baseline, during the intervention (6 weeks following recruitment), at the end of the intervention group (12 weeks following recruitment), and 3 months after completing the intervention. During each visit, a series of assessments were conducted:

1. Functional performance assessment:
  The 6MWT, assessing endurance and functional capacity at every visit, as well as STST and HST to evaluate physical endurance and exercise intolerance, providing insight into overall muscle strength and physical resilience.
  For safety reasons, every participant received an activity tracker (Garmin Vivo Smart 5 https://www.garmin.com/de-DE/pri-vacy/connect/policy/) measuring heart rate and status of activity during the weeks of intervention to enable the timely identification of worsening vital signs daily.
2. Physiological assessments:
  Participants underwent laboratory tests and clinical examinations, including assessments by interdisciplinary pediatric specialists in neurology, pulmonology, cardiology, and infectious diseases to monitor overall health at every visit as stated in supplement 1 (relevant variables for this study are underlined). All patient received at least two instrument-based diagnostics including measurements of vital capacity (VC), electrocardiography (ECG), echocardiography, ultrasound of the abdomen and thorax, 24-h electrocardiography, and blood pressure measurements. When clinically indicated, further investigations and other pediatric relevant specialties were included.
3. Psychological assessments:
  Patients and their parents were requested to fill out different questionnaires, including the PedsQL^TM^ about life quality and fatigue during every visit. A therapy evaluation questionnaire (GEEE) was completed in the 2nd, 3^rd^, and 4th visits (see supplement 3).Throughout the 12-week study, participants maintained an online diary to record their symptoms, participation in activities, daily school attendance, and hobbies. Each participation was reviewed with a pediatric and adolescent psychologist to ensure adequate mental stability and to provide support if needed (see also supplement 2–4).

### Individualized online exercise therapy (IOET)

The patients followed an IOET with sports therapists of the IST-University of Applied Sciences, Duesseldorf twice a week. The sports therapists were supervised by experienced therapists specialized in pediatric oncology patients. Therapy sessions were individualized based on the symptoms and the possible activities of the participants following an evidence-based framework [21]. Please see also supplement 6. To ensure standardization of sessions carried out by different therapists, weekly meetings were scheduled between the therapist, nurses, and physicians involved in the study. These meetings were dedicated to planning each session for each patient and summarizing all documented symptoms, complaints, and wishes of the patients, as recorded in their diaries and presented in the questionnaires.

All patients received individualized handouts for carrying on with their exercises by themselves after completing the intervention at the end of the IOET program as shown in Fig. 2.

The dataset comprised physical exam, laboratory tests, and diagnostic procedures conducted during the four scheduled visits at the long-COVID outpatient clinic. In addition, participants and their parents completed quality-of-life and treatment expectancy questionnaires, along with maintaining participant diaries collected electronically via Limesurvey.

### Statistical analysis

Descriptive statistics were used to summarize demographic and clinical characteristics within each group. Continuous variables were reported as means with standard deviations (SD), while categorical variables were summarized using frequencies and percentages.

Mean values and 95% confidence intervals for the 6MWT, STST, and HST were calculated separately for each group across all four visits. Questionnaire responses were analyzed to compare groups, and mean changes in perceived improvement and reported side effects were computed. All other statistical analyses were performed using Microsoft Excel, IBM SPSS Statistics, and R (version 27).

A linear mixed-effects model was used to assess the effects of time, group, and their interaction on the outcome variables. This approach accounts for within-participant correlations. The model was fit and analyzed using the lme4 and lmerTest [23, 24].

The main covariates of interest were modeled as fixed effects: group (6 weeks vs. 12 weeks or IOET) and visit number, representing within-subject change. An interaction term between visit and group was included to evaluate differential temporal trajectories. A random intercept was included. Additional covariates, such as sex and age, were not included in the model, and the overall sample size was limited to avoid overfitting and ensure model stability.

Linear mixed-effects models assume linearity, normally distributed residuals with constant variance, and independence after accounting for random effects. These assumptions would likely hold in a larger population but were not formally tested due to the small sample size. Given the small sample size and the high variability in the data, we focused on descriptive reporting rather than emphasizing *p*-values. To provide transparency, a table including *p*-values and 95% confidence intervals has been added in the Supplemental Materials. As this was an exploratory study, *p*-values were not adjusted for multiple testing.

Little’s MCAR test indicated that the data were missing completely at random. Missing values were subsequently imputed using a LASSO-based regression approach [22]. Cohen’s *d* was calculated as the estimated difference between groups divided by the residual standard deviation. As Cohen’s *d* is not formally defined for longitudinal mixed-effects models, it was computed only for the primary endpoint (Visit 3).

## Ethics approval

The study was conducted in accordance with the Declaration of Helsinki. The Ethics Committee of the Medical Faculty of the University of Duisburg-Essen approved the study (22–11168-BO). Patient and parental informed consent was necessary, and patient’s and legal guardians’ written informed consent was given to participate. The clinical trial was registered in the German register of clinical trials (DRKS00034939). Since online data collection and telemedicine procedures were utilized, all data were anonymized prior to analysis in compliance with the General Data Protection Regulation (GDPR, Regulation (EU) 2016/679). Data storage and processing followed institutional and legal standards for data protection and confidentiality.

## Results

A total of 14 patients (3 males, 11 females) with a median age of 14.6 years (range 9–17 years) were included in the study. Two patients were excluded prior to enrolment due to severe psychiatric comorbidities requiring psychiatric inpatient care. One additional eligible patient declined participation, as illustrated in Fig. 1 and supplement 7.1 table S1.

The most frequently reported baseline symptoms were fatigue, recurrent headaches, concentration difficulties, and increased need for sleep, as shown in Table 2 and supplement 7.2–3 table S2 and table S3. The mean duration of symptoms prior to inclusion was 21 months. Pre-existing conditions among the cohort included congenital myotonia (*n* = 2), bronchial asthma (*n = *2), diagnosed and treated depression (*n* = 2), migraine (*n* = 1), and one case with hyperCKaemia of unknown etiology. One participant was a competitive athlete (supplement 7.1 table S1).

**Table 2:** Patient characteristics

		Group 1 (12 weeks IOET)	Group 2 (6 weeks IOET)
		*n* = 7	*n* = 7
Male		1 (14.3%)	2 (28.6%)
Age (years)	Mean (95% CI)	15.3 (12–17)	14.0 (9–17)
Time since diagnosis of LC (months)	Mean (95% CI)	19.4 (13–26)	16.0 (8–24)
Previous health issues	Congenital myotonia, Type Thompson	1 (14.3%)	1 (14.3%)
	Bronchial asthma	1 (14.3%)	1 (14.3%)
	Diagnosed and treated depression	1 (14.3%)	1 (14.3%)
	Accommodation disorder/migraine	0 (0%)	1 (14.3%)
	Congenital elevated CK	1 (14.3%)	0 (0%)
Relevant additional information	High-performance athlete	1 (14.3%)	0 (0%)
Symptoms			
	Duration until inclusion in the study month (mean)	13–33 (24)	11–31 (19)
	Concentration difficulties	5 (71.4%)	7 (100%)
	Fatigue	7 (100%)	7 (100%)
	Asthma	2 (28.6%)	1 (14.3%)
	Stomach pain	1 (14.3%)	1 (14.3%)
	Myocarditis*	1 (14.3%)	0 (0%)
	Amenorrhoea**	1 (16.7%)	0 (0%)
	Weight loss	1 (14.3%)	1 (14.3%)
	Hair loss	1 (14.3%)	0 (0%)
	Paraesthesia	5 (71.4%)	7 (100%)
	Vertigo	4 (57.1%)	4 (57.1%)
	Headache	1 (14.3%)	5 (71.4%)

*AWMF*, German Association of the Scientific Medical Societies; *NICE*,  National Institute for Health and Care Excellence; * one patient had suffered from an initial myocarditis, which was asymptomatic, and due to a normal cardiac MRI, she was allowed to participate within the study according to her pediatric cardiologists, **n = 6

Overall compliance with the intervention was high: 85% of all scheduled sessions were attended, with missed sessions primarily due to illness or technical issues. Group 2 completed 91.7% of sessions (*n* = 72), while group 1 completed 76.4% (*n* = 144) (supplement 7.1 table S1).

## Primary outcomes

Physical performance was assessed using the 6MWT, STST, and HST. Improvements were observed across all measures from baseline (visit 1) to post-intervention (visit 3) (supplement 7.1 table S1).

After 12 weeks of IOET, participants in group 1 showed marked improvements across all performance measures (Fig. 2). The 6MWT increased from a mean distance of 396.0 m (95% CI: 286.1–505.9) to 616.3 m (380.5–852.1). Similarly, the 30-s STST improved from 25.4 repetitions (14.9–36.0) to 32.6 repetitions (13.7–51.4), and HST rose from 16.6 kg (9.4–23.7) to 27.1 kg (19.1–35.2).

**Fig. 3:**
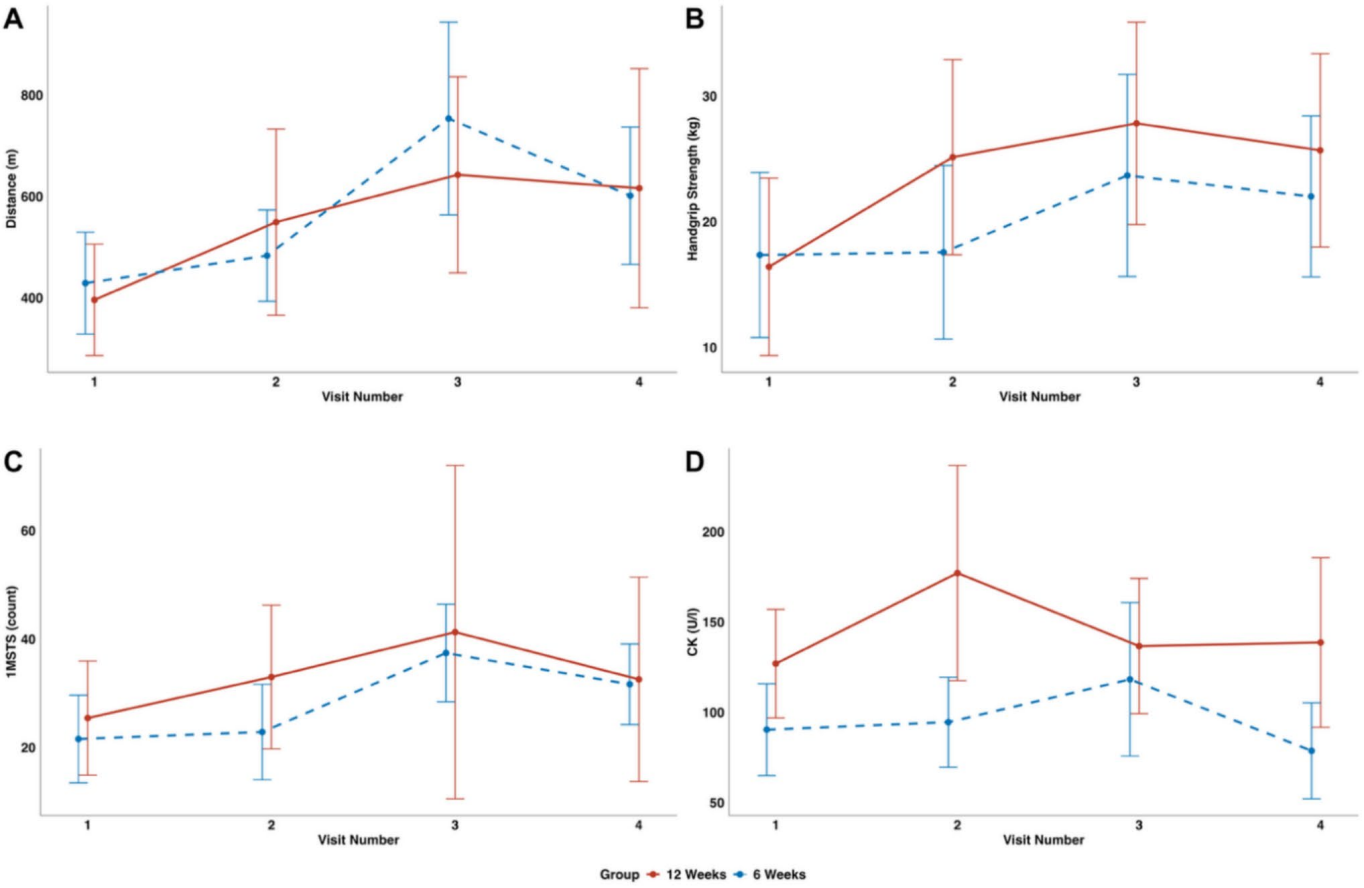
Changes in functional performance (6MWT, HST, STST) and serum creatine kinase levels over time within group 1 (red—IOET from visit 1 to 3) and group 2 (blue—IOET only from visit 2 to 3). **A** 6-Minute walk test (y-axis (meters walked in 6 min) and the x-axis (time across visits 1 to 4)); **B** handgrip (y-axis (median handgrip strength test for both hands, measured in kilograms, assessed over three trials) and the x-axis (time across visits 1 to 4)); **C** sit-to-stand test (y-axis (rounds in 1 min) and the x-axis (time across visits 1 to 4)); **D** serum creatinine kinase (CK) during intervention (y-axis (CK U/l) and the x-axis (time across visits 1 to 4)); *n* = 14 (group 1 *n* = 7 and group 2 *n* = 7). For additional information, the 95% CIs are added: **A** 6MWT—group 1: 396.0 m (95% CI 286.1–505.9) at visit 1 vs. 616.3 m (95% CI 380.5–852.1) at visit 4; group 2: 429.0 m (95% CI 328.6–529.5) vs. 601.6 m (95% CI 466.4–736.8). **B** Handgrip—group 1: 16.6 kg (95% CI 9.4–23.7) at visit 1 vs. 27.1 kg (95% CI 19.1–35.2) at visit 4; group 2: 17.3 kg (95% CI 10.9–23.7) vs. 22.1 kg (95% CI 15.9–28.4). **C** Sit-to-stand test—group 1: 25.4 repetitions (95% CI 14.9–36.0) at visit 1 vs. 32.6 (95% CI 13.7–51.4) at visit 4; group 2: 21.6 (95% CI 13.5–29.7) vs. 31.7 (95% CI 24.2–39.1). **D** CK—group 1: 127.0 U/l (95% CI 97.0–157.0) at visit 1 vs. 138.7 U/l (95% CI 91.8–185.7) at visit 4; group 2: 90.4 U/l (95% CI 65.0–115.9) vs. 78.7 U/l (95% CI 52.1–105.3). For illustrative purposes, Cohen’s *d* was calculated for visit 3, yielding values of 0.48 for handgrip strength, −0.54 for the 6-minute walking test, 0.43 for CK, and 0.16 for the sit-to-stand test. For patient-reported outcomes, Cohen’s *d *values were −0.22 and 0.00 for quality of life, and −0.41 and −0.27 for fatigue (patients and parents, respectively). These estimates should be interpreted with caution

In group 2, which completed 6 weeks of IOET, comparable positive trends were observed. The 6MWT improved from 429.0 m (328.6–529.5) to 601.6 m (466.4–736.8), the STST increased from 21.6 repetitions (13.5–29.7) to 31.7 repetitions (24.2–39.1), and HST rose from 17.3 kg (10.9–23.7) to 22.1 kg (15.9–28.4). Although intergroup differences (12 week vs. 6-week intervention) were not statistically significant, group 1 demonstrated early improvements between visits 1 and 2, aligning with the earlier onset of therapy (Fig. 1 A–D and supplement 7.1 table S1). Individual baseline variability was noted. At 3-month follow-up (visit 4), physical performance declined compared to visit 3 but remained above baseline levels (Fig. 3A–D and supplement 7.1–3 table S1 to S3).

## Secondary outcomes

To assess potential adverse effects of the intervention, routine laboratory tests were performed (supplement 1). There were no relevant differences between the groups. No relevant abnormalities were observed in cardiac enzymes or urinary myoglobin levels. CK levels increased during the early intervention phase but remained within safe limits and were not associated with clinical signs of rhabdomyolysis. CK levels subsequently declined despite continued physical activity (Fig. 1 and supplement 7.1–2 table S1 and S2). Intra-group differences in CK were seen due to high baseline variability, particularly at visit 2 (group 1) and visit 3 (group 2), though no between-group differences were noted (supplement 7.3 table S3).

Prior to the intervention, the average face-to-face school attendance rate for everyone was 58% (SD ± 23.8). This increased to 97% (SD ± 10.7) by visit 3 (both groups) after the completion of exercise therapy. Additional engagement in sports activities outside of the study was reported more frequently after the intervention compared to baseline (50% der patients).

**Fig. 4:**
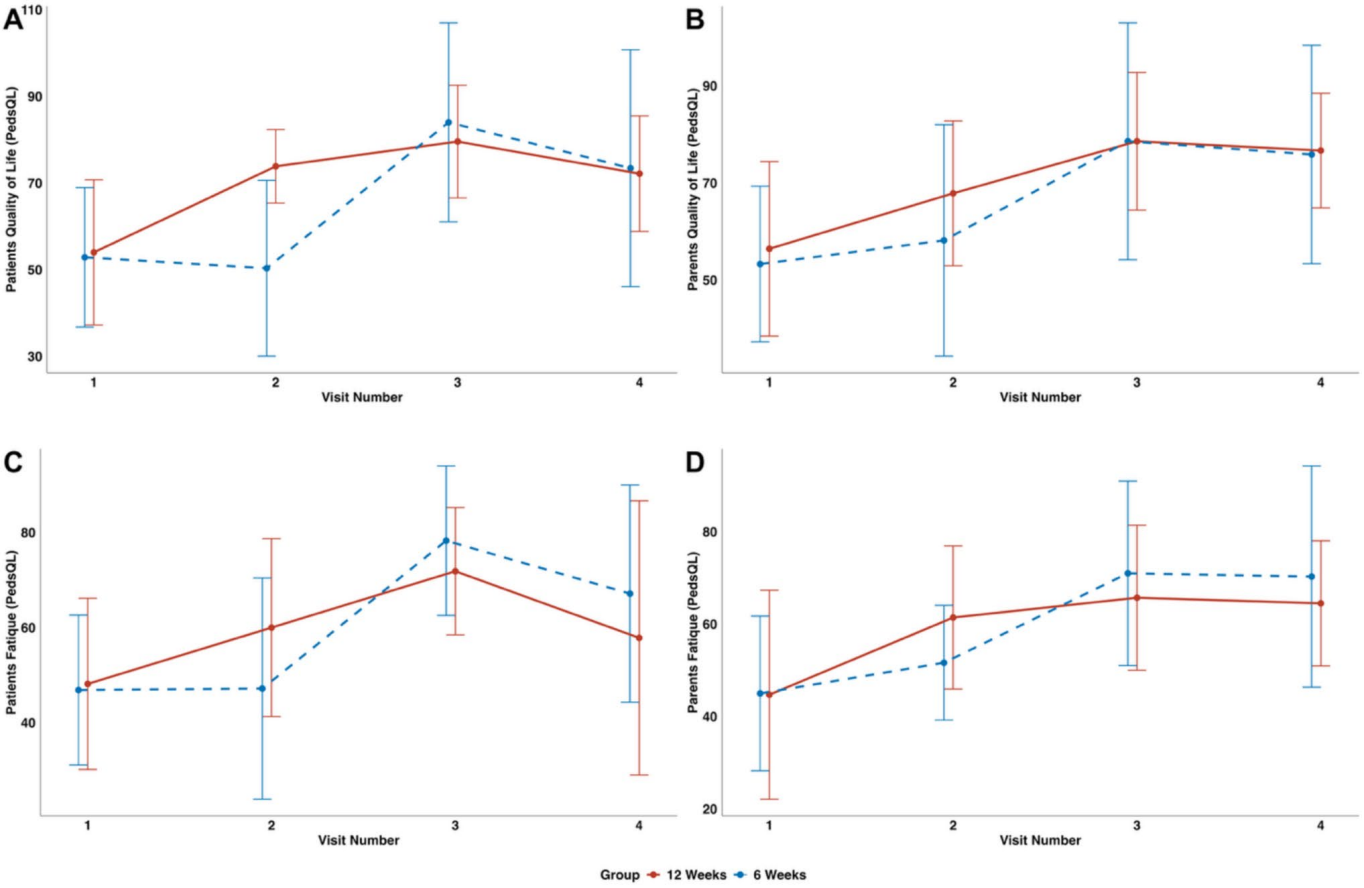
Effect of overall exercise therapy on the health-related quality of life and fatigue, assessed by PedsQL. **A** Quality of life assessed by patients (y-axis (PedsQL total score) and the x-axis (time across visits 1 to 4)); **B** quality of life assessed by parents (y-axis (Peds QL total score) and the x-axis (time across visits 1 to 4)); **C** fatigue assessed by patients (y-axis (PedsQL total score) and the x-axis (time across visits 1 to 4)); **D** fatigue assessed by parents (y-axis (PedsQL total score) and the x-axis (time across visits 1 to 4)); *n* = 14 (group 1 *n = *7 and group 2 *n* = 7). For additional information, the 95% CIs are added: **A** Quality of life (patients)—model intercept: 54.0 (95% CI 39.26–68.74); change at visit 3: 25.57 (95% CI 12.69–38.46). **B** Quality of life (parents)—model intercept: 56.43 (95% CI 41.05–71.81); change at visit 3: 22.14 (95% CI 8.66–35.63). **C** Fatigue (patients)—model intercept: 48.14 (95% CI 32.62–63.66); change at visit 3: 23.71 (95% CI 11.30–36.13). **D** Fatigue (parents)—model intercept: 44.71 (95% CI 29.92–59.51); estimated change at visit 3: 21.00 (95% CI 8.89–33.11)

Using the PedsQL™, patients and parents reported improvements in all domains, including physical, emotional, social, and school functioning (Fig. 4 and supplement 7.1 table S1). Fatigue scores, encompassing general fatigue, need for rest, and mental fatigue, also improved following the intervention (Fig. 4 and supplement 7.1 table S1). Although scores decreased slightly at the 3-month follow-up, they remained elevated compared to baseline values (Fig. 4 and supplement 7.1 table S1). For patient-reported QoL, we found a group and time interaction: Visit 2 shows an interaction between the 6 weeks group and the reference group. Specifically, the 6 weeks group shows a smaller change from baseline at visit 2.

Patients and parents rated the perceived effect of the exercise therapy on a 10-point scale, with a mean score of 8 from patients (SD ± 1.3) and 7 from parents (SD ± 2.4) (supplement 7.3 table S3). No significant psychological deterioration attributable to the intervention was reported (mean deterioration score: 0 by parents, 1 by patients).

At completion of the study, 9 out of 14 participants (64%) reported full recovery from long-COVID and opted out of further follow-up, despite occasional residual symptoms after completion of the study. One additional patient completed only the final questionnaire and reported complete resolution of symptoms.

Additional participation in sports activities outside the study increased after the intervention, indicating a rise in overall physical activity and confidence. A total of 50% of participants restarted sports like tennis, gymnastics, and dance or engaged in new sports outside of school.

One participant in the 6-week intervention group (patient 4) exhibited primarily physical improvement, with minimal change in school attendance and psychosocial functioning. Ongoing symptoms of low mood and reduced energy were noted in diaries and questionnaires from week 1 to week 4, prompting referral for psychiatric evaluation and further therapy.

## Discussion

This study demonstrates the beneficial effects of individualized online exercise therapy on physical capacity, quality of life, and school participation in pediatric patients with long-COVID. The improvements in 6MWT, STST, and HST and a 39% increase in school attendance highlight its therapeutic potential. However, these findings must be interpreted with caution due to the exploratory nature of the study and the small sample size.

All participants under IOET experienced clinically meaningful improvements. As reported by one parent in the GEEE questionnaire, “My child is finally attending school again, something we feared might never happen.” While such subjective qualitative statements illustrate individual benefit, their use should remain limited and interpreted as supportive rather than primary evidence of efficacy.

Long-COVID manifests with a broad and heterogeneous range of symptoms, with fatigue among the most frequent, as reported by Pellegrino et al. [25]. The challenge of managing this complex symptomatology is reflected in emerging literature debating phenotyping strategies, yet the need for therapeutic options remains pressing. Organowska-Slodownik et al. and the WHO emphasize that structured rehabilitation programs are among the few available interventions for pediatric long-COVID [26, 27].

Exercise therapy has long been established as beneficial across chronic diseases, improving not only physical outcomes but also social functioning and emotional resilience [28]. Numerous studies report benefits of exercise in populations with cancer, post-surgical fatigue, chronic respiratory conditions, and even lower mortality [13, 29–31]. Spreafico et al. reported “organized physical exercise reduced fatigue and improved HRQoL in children with cancer” [32].

In this study, an exercise program designed for pediatric oncology patients was adapted for long-COVID, supplemented by “Physical Activity-Related Health Competence” (PAHCO), emphasizing motivation, progression, and individualization (supplement 3). As one therapist reflected during the weekly planning meetings, “When children feel seen and supported, they’re much engaged even on tough days” [22].

Concerns that exercise might worsen symptoms, especially in children with myalgic encephalomyelitis/chronic fatigue syndrome (ME/CFS) or post-exertional malaise (PEM), are understandable. However, no participant reported symptoms of deterioration, even though at least three met the Canadian ME/CFS criteria retrospectively. Even patients with neuromuscular comorbidities (e.g., myotonia congenita) completed the program safely. One patient reported in their diary: “I was afraid the training would make me feel worse. But instead, I feel like I got my strength back.” This supports findings by Larun and Fugazzaro et al. on safe implementation in chronic fatigue [33, 34]. Nonetheless, generalizability to more severely affected children (Bell CFIDS < 30) is uncertain and requires further study.

Close medical supervision and the use of activity trackers played a key role in ensuring patient safety throughout the program. These safety strategies align with best practice recommendations [35]. As a result, no adverse events such as cardiac issues or a physical “crash” occurred.

Telemedicine was crucial for implementation, as many participants lived more than 2 h distance by car from the clinic. Online delivery ensured access and high attendance (> 85%). Previous research confirms that online exercise programs are feasible, well-accepted, and potentially cost-saving in chronic pediatric conditions [36, 37].

IOET therefore offers a clinically scalable rehabilitation model that can be integrated into pediatric rehabilitation services, delivered remotely through secure telehealth platforms, and used as a structured clinical guidance framework for clinicians treating fluctuating long-COVID symptoms. IOET aligns with the 2024 WHO pediatric long-COVID rehabilitation statements, which emphasize individualized activity pacing, interdisciplinary care models, and accessible digital rehabilitation pathways to support participation in education and daily life [27].

As Davis et al. have emphasized, more prospective randomized controlled trials are needed to evaluate treatment strategies for long-COVID [38]. Our results support efficacy based on 6MWT and validated questionnaires. The question of optimal therapy duration remains open. Riggs et al. found 12weeks beneficial in adults, while our 6-week program also showed relevant improvements [39].

Spontaneous remission of long-COVID symptoms is possible, but unlikely to explain the observed changes in our cohort, where the median symptom duration was 21 months before inclusion [40]. Organowska-Slodownik et al. similarly reported improvements after 8 + 4 weeks [26].

Sustained benefit at 3 months suggests a lasting effect. However, 3 months of follow-up represents a relatively short observation period, and even within this limited timeframe, performance metrics like walking distance and strength declined slightly after the end of the intervention. This implies sustainability may require continued activity. Long-term studies should assess how to preserve improvements.

Baseline values for CK were heterogeneous within the normal range of the general population. Given that there was no healthy control group and some sex-related imbalances (e.g., a higher proportion of boys in one arm), it is difficult to fully attribute changes in CK to the intervention. However, there were no test results suggesting a harmful rise.

A highly relevant aspect deserving further exploration is psychological support. Mat Hassan et al. reported high rates of anxiety, depression, and mood disturbances in children with long-COVID [9]. Four participants required psychiatric care. Psychological modules may enhance future rehabilitation outcomes.

Importantly, IOET was safe, feasible, and effective in improving mental and physical well-being, including strength, quality of life, and participation in daily life activities like school attendance. As one adolescent expressed at the study’s conclusion: “It feels like I finally have a way back into my life.” Yet, because the study was not powered for confirmatory conclusions, the findings should be interpreted as preliminary and hypothesis-generating rather than definitive evidence. These results warrant confirmation in larger, multicenter trials and support individualized online training as a viable therapeutic pathway in the multidisciplinary care of pediatric long-COVID.

## Limitations

This study is limited by its small sample size, single-center and exploratory design, and the absence of cognitive assessments. The lack of a no-intervention control group restricts causal attribution. Limited participant diversity, minimal therapist variation, and a follow-up period of only 3 months may also have introduced bias. Nevertheless, consistent improvements across outcomes support the feasibility and potential benefit of the intervention.

## Conclusion

Individualized online exercise therapy appears safe, feasible, and potentially beneficial in the multidisciplinary care of pediatric long-COVID, particularly regarding physical function and school participation. However, due to the exploratory design and small sample size, the findings cannot be generalized. Larger trials are needed to determine optimal treatment duration, long-term effects, and suitability for patients with severe PEM or complex comorbidities.

## Supplementary Information

Below is the link to the electronic supplementary material.

ESM 1 (DOCX 100 KB)

## Data Availability

No datasets were generated or analysed during the current study.

## Abbreviations

ANA/ANCA: Anti-nuclear/anti-neutrophil cytoplasmic antibodies
ATS, ERS: American Thoracic Society/European Respiratory Society
AWMF: Association of Scientific Medical Societies (German: Arbeitsgemeinschaft der Wissenschaftlichen Medizinischen Fachgesellschaften e. V., abbreviated AWMF)
BP: Blood pressure
CI: Confidence interval
CK: Creatine kininase
CK-MB: Creatine kinase myocardial band
CRP: C-reactive protein
CT: Computed tomography (falls nicht genutzt: streichbar)
EEG: Electroencephalogram
ECG: Electrocardiogram
ESR: Erythrocyte sedimentation rate
GEE-E: Therapy evaluation questionnaire
HR: Heart rate
HRQoL: Health related quality of life
HST: Handgrip strength test
IgA/M/G/E: Immunoglobulin subtypes
IOET: Individualized online exercise therapy
IST: Institute for sport and touristic
kg: Kilograms
LC: Long-COVID
LDH: Lactate dehydrogenase
LoCoSpo: Long-COVID sport
ME/CFS: Myalgic encephalomyelitis/chronic fatigue syndrome
MRI: Magnetic resonance imaging
NASEM: National Academies of Sciences, Engineering, and Medicine
NICE: National Institute for Health and Care Excellence
NT-proBNP: N-terminal pro brain natriuretic peptide
P: Probability value
PACS: Post-acute sequelae of severe acute respiratory syndrome coronavirus 2 infection
PAHCO: Physical activity-related health competence
PCR: Polymerase chain reaction
PedsQLTM PedsQL: Life quality questionnaire
PEM: Post-exertional malaise
PT: Prothrombin time
SD: Standard deviation
SpO₂: Peripheral oxygen saturation
STST: Sit-to-stand test
TSH, fT4: Thyroid parameters
U/l: Units/liter
VC: Vital capacity
WHO: World Health Organization
6MWT: 6-Minute walking test

## Units of measure

6-Minute walking test: M
Cortisol: Nmol/l
C-reactive protein: Mg/dl
creatine kinase: U/l
Lactate: Mmol/l
Participation at school: %
SARS-CoV-2-IgG: BAU/ml
Sit-to-stand test: 1/Minute
Handgrip strength test: Weight in kg
Troponine I: Ng/l
Urinary myoglobin: µG/ml
Vital capacity: %
