# Supplementary material for: Individualized online exercise therapy aids recovery in pediatric long-COVID—findings from an exploratory randomized controlled trial
Source: Eur J Pediatr. 2026 Jan 6;185(1):54. doi: 10.1007/s00431-025-06705-5 (PMC12774993; doi:10.1007/s00431-025-06705-5)
Supplement: Supplementary file 1 — (DOCX 100 KB) [file 431_2025_6705_MOESM1_ESM.docx]

**Supplement:**

1. **Supplement 1 – Standard diagnostic workup performed for every patient:**

- **Laboratory Diagnostics:**
  Comprehensive blood testing was performed, including: complete blood count with differential, coagulation parameters (PT, INR, aPTT, thrombin time, fibrinogen, antithrombin III, D-dimer), serum electrolytes (including potassium and phosphate), renal function markers (creatinine, cystatin C, urea), cardiac markers (CK, CK-MB, troponin T, myoglobin, NT-proBNP), liver enzymes (transaminases, LDH), inflammatory parameters (CRP), lipid status (triglycerides), complement factors (C3, C4), haptoglobin, immunoglobulins (IgA, IgM, IgG, IgE), erythrocyte sedimentation rate, blood gas analysis, endocrine parameters (cortisol, TSH, fT4), SARS-CoV-2 IgG, 25-OH vitamin D, vitamin B1, B6 and B12 levels, folate, serology for coeliac disease, ferritin, ANA, ANCA, and serotonin.
  Urine diagnostics included: urinalysis with microscopy and 24-hour urinary cortisol.
  The laboratory workup was expanded in a symptom-oriented manner.
- **Standardised Clinical Parameters:**
  Body weight, height, heart rate, blood pressure, and oxygen saturation were recorded.
- **Standardised Diagnostic Procedures:**
  Echocardiography, 12-lead ECG, long-term ECG monitoring, ambulatory blood pressure measurement, pulmonary function testing, abdominal and thyroid ultrasonography, and EEG. If clinically indicated, additional diagnostics included nerve conduction studies and MRI of the brain, spine, or heart.
- **Specialist Consultations:**
  Depending on symptomatology, consultations with child and adolescent psychiatry, otorhinolaryngology, gynaecology and other specialties were obtained as required.

1. **Supplement 2 – Questionnaires: Health-Related Quality of Life and Fatigue (PedsQL™) Questionaires**

**(All questionnaires used in this study have been previously validated; Those shown here are examples for age 7-12 years)**

**2.1 PedsQL – Parent proxy report (example: 7–12 years) (**[**https://www.pedsql.org**](https://www.pedsql.org)**)**

Version 4.0 - Short Form (SF15) – German (Germany)

**Has your child had the following problems or difficulties in the PAST 4 WEEKS?**

**PROBLEMS WITH HEALTH AND ACTIVITIES**

My child…

never Rarely sometimes often Almost always ...

had trouble walking more than 100 meters. 0 1 2 3 4 ...

had trouble running. 0 1 2 3 4 ...

had trouble participating in sports activities. 0 1 2 3 4 ...

had trouble lifting something heavy. 0 1 2 3 4 ...

had trouble doing household chores. 0 1 2 3 4

**PROBLEMS WITH MOOD/EMOTIONS**

My child…

never Rarely sometimes often Almost always ...

felt afraid. 0 1 2 3 4 ...

was sad. 0 1 2 3 4 ...

was angry. 0 1 2 3 4 ...

worried about what would happen to him/her. 0 1 2 3 4

**PROBLEMS IN INTERACTIONS WITH OTHER YOUTH**

never Rarely sometimes often Almost always ...

had trouble getting along with other children. 0 1 2 3 4 ...

had problems because other children did not want to be friends with him/her 0 1 2 3 4 ...

was teased by other children. 0 1 2 3 4

**PROBLEMS AT SCHOOL**

never Rarely sometimes often Almost always ...

had trouble paying attention in class. 0 1 2 3 4 ...

forgot things. 0 1 2 3 4 ...

had trouble completing schoolwork and tasks. 0 1 2 3 4

**PROBLEMS WITH GENERAL FATIGUE**

My child…

never Rarely sometimes often Almost always ...

felt tired. 0 1 2 3 4 ...

felt physically weak (not strong). 0 1 2 3 4 ...

felt too tired to do things they like. 0 1 2 3 4 ...

felt too tired to spend time with friends. 0 1 2 3 4 ...

had trouble finishing things they started. 0 1 2 3 4 ...

had trouble starting things. 0 1 2 3 4

**PROBLEMS WITH FATIGUE / NEED FOR REST**

My child…

never Rarely sometimes often Almost always ...

slept a lot. 0 1 2 3 4 ...

had trouble sleeping through the night. 0 1 2 3 4 ...

felt tired when they woke up in the morning. 0 1 2 3 4 ...

rested a lot. 0 1 2 3 4 ...

took many naps. 0 1 2 3 4 ...

spent a lot of time in bed. 0 1 2 3 4

**PROBLEMS WITH MENTAL FATIGUE**

My child…

never Rarely sometimes often Almost always ...

had trouble focusing on things. 0 1 2 3 4 ...

had trouble remembering what people told them. 0 1 2 3 4 ...

had trouble remembering things they just heard. 0 1 2 3 4 ...

had trouble thinking quickly. 0 1 2 3 4 ...

had trouble remembering what they just thought. 0 1 2 3 4 ...

had trouble remembering more than one thing at a time. 0 1 2 3 4

**2.2 PedsQL^TM^ – Patients self-report (adolescents) (example: 7–12 years) (**[**https://www.pedsql.org**](https://www.pedsql.org)**)**
Version 4.0 - Short Form (SF15) – German (Germany) QUESTIONNAIRE for ADOLESCENTS

**In the past 4 WEEKS, did you experience the following problems or difficulties?**

**HEALTH AND ACTIVITY PROBLEMS**
Never Almost Never Sometimes Often Almost Always
It was hard for me to walk more than 100 meters. 0 1 2 3 4
It was hard for me to run. 0 1 2 3 4
It was hard for me to participate in sports activities. 0 1 2 3 4
It was hard for me to lift something heavy. 0 1 2 3 4
It was hard for me to do household chores. 0 1 2 3 4

**MOOD/FEELING PROBLEMS**
Never Almost Never Sometimes Often Almost Always
I felt afraid. 0 1 2 3 4
I was sad. 0 1 2 3 4
I was angry. 0 1 2 3 4
I worried about what would happen to me. 0 1 2 3 4

**PROBLEMS WITH OTHER ADOLESCENTS**
Never Almost Never Sometimes Often Almost Always
I had trouble getting along with other kids. 0 1 2 3 4
Other kids didn’t want to be friends with me. 0 1 2 3 4
Other kids teased me. 0 1 2 3 4

**PROBLEMS AT SCHOOL**
Never Almost Never Sometimes Often Almost Always
I had trouble paying attention in class. 0 1 2 3 4
I forgot things. 0 1 2 3 4
I had trouble completing schoolwork and tasks. 0 1 2 3 4

**GENERAL EXHAUSTION PROBLEMS**
Never Almost Never Sometimes Often Almost Always
I feel tired. 0 1 2 3 4
I feel physically weak (not strong). 0 1 2 3 4
I feel too tired to do things I like. 0 1 2 3 4
I feel too tired to spend time with friends. 0 1 2 3 4
I have trouble finishing things I start. 0 1 2 3 4
I have trouble starting things. 0 1 2 3 4

**FATIGUE/NEED FOR REST PROBLEMS**
Never Almost Never Sometimes Often Almost Always
I sleep a lot. 0 1 2 3 4
I have trouble sleeping through the night. 0 1 2 3 4
I feel tired when I wake up in the morning. 0 1 2 3 4
I rest a lot. 0 1 2 3 4
I take a lot of naps. 0 1 2 3 4
I spend a lot of time in bed. 0 1 2 3 4

**MENTAL FATIGUE PROBLEMS**
Never Almost Never Sometimes Often Almost Always
I have trouble keeping my attention focused. 0 1 2 3 4
I have trouble remembering what people tell me. 0 1 2 3 4
I have trouble remembering things I just heard. 0 1 2 3 4
I have trouble thinking quickly. 0 1 2 3 4
I have trouble remembering what I was just thinking. 0 1 2 3 4
I have trouble remembering more than one thing at a time. 0 1 2 3 4

1. **Supplement 3 – Therapy Effects: GEEE Current Effects (Parent and Patient)**

**3.1 Instrument description**

**Generic rating scale for previous treatment experiences, treatment expectations, and treatment effects (GEEE).**

**Source: Rief W, Nestoriuc Y, Mueller EM. Generic rating scale for previous treatment experiences, treatment expectations, and treatment effects (GEEE). PsychArchives 2021. doi:10.23668/psycharchives.4717**

**Scale: numeric rating scales from 0–10.**

**3.2 Parent version – current therapy effects (GEEE Current Effects)**

In the following section, please describe any changes your child has experienced since participating in this study, based on your perception:

1. How much improvement in symptoms has your child experienced since then?
   No improvement 0 1 2 3 4 5 6 7 8 9 10 Maximum imaginable improvement
2. How much worsening of symptoms has your child experienced since then?
   No worsening 0 1 2 3 4 5 6 7 8 9 10 Maximum imaginable worsening
3. How many complaints/side effects from the treatment has your child experienced since then?
   No complaints 0 1 2 3 4 5 6 7 8 9 10 Maximum imaginable complaints

**3.3 Patient version – current therapy effects (GEEE Current Effects)**

In the following section, please describe any changes you have experienced since participating in this study:

1. How much improvement in symptoms have you experienced since then?
   No improvement 0 1 2 3 4 5 6 7 8 9 10 Maximum imaginable improvement
2. How much worsening of symptoms have you experienced since then?
   No worsening 0 1 2 3 4 5 6 7 8 9 10 Maximum imaginable worsening
3. How many complaints/side effects from the treatment have you experienced since then?
   No complaints 0 1 2 3 4 5 6 7 8 9 10 Maximum imaginable complaints
4. **Supplement 4 – Daily Online Diary (Patient-Reported Outcomes)**

To help us understand how you are feeling during the study, we ask you to fill out the first part daily and the second part after your training sessions. The diary consisted of:

**4.1 Daily symptom and functioning section**
Never Almost Never Sometimes Often Almost Always
It was hard for me to walk more than 100 meters. 0 1 2 3 4
I feel physically weak (not strong). 0 1 2 3 4
I need to rest a lot. 0 1 2 3 4
I have trouble concentrating. 0 1 2 3 4

If you felt good today, you don't need to answer this question. If you didn’t feel well, list your top 3 symptoms. Please name the symptom that bothered you the most first.
**Free text:**
I went to school today: Yes, No

**How long did you go to school in hours:**
Other (e.g., I was sick today): **Free text**
I had was physical active today: Yes No

What did you do: Free text

Do you want to tell us something: Free text

**4.2 Training-day section (only on exercise therapy days)**

**4.2.1 The flowering were only answered on exercise therapy days by the patient:**
What did you like about the training session, and did you enjoy it? What didn't you like: **Free text:**Do you feel that your symptoms have improved compared to the last training session: **Free text:**Do you have any wishes for the next training session: **Free text**Did you exercise independently between training sessions: Free text
If yes, how often and for how long? What did you do**: Free text**
Did you use an activity tracker: Yes, No

**If No –** Why: Free text

**4.2.2 The flowering were only answered on exercise therapy days by the physical therapist:**
Were there any technical problems?
Was everyone on time?
Break times?
Unplanned events/incidents?

1. **Supplement 5 – Functional Performance Assessments (Primary and Secondary Outcomes)**

| **Test** | **Method** | **Which aspect of physical resilience was specifically assessed?** | **References** |
| --- | --- | --- | --- |
| 6MWT | The 6-minute walk test (6MWT) was performed according to the American Thoracic Society (ATS) guidelines. All tests were conducted in the same indoor corridor, which was 6 meters long and measured in advance. Participants walked continuously in a loop along this predefined course to ensure a consistent walking distance and environment. The test was performed individually to avoid interruptions or distractions. Standardized verbal encouragement was provided every minute by the assessor from outside the walking area. Before the test, baseline heart rate (HR), oxygen saturation (SpO₂), blood pressure (BP), Borg dyspnoea, and perceived exertion were recorded. Participants were instructed to walk as far as possible for six minutes, being allowed to slow down or stop if necessary but encouraged to resume walking as soon as they could. Immediately after the test, the total distance covered (in meters) was documented. HR, SpO₂, BP, dyspnoea, and exertion were reassessed at 0-, 1-, 3-, and 5-minutes post-test. As well was the distance measured walked after 6 minutes. | Endurance-based resilience (functional recovery capacity) | American Thoracic Society. ATS statement: guidelines for the six-minute walk test. Am J Respir Crit Care Med. 2002;166(1):111–117. doi:10.1164/ajrccm.166.1.at1102  Geiger R, Strasak A, Treml B, Gasser K, Kleinsasser A, Fischer V, Geiger H, Loeckinger A, Stein JI. Six-minute walk test in children and adolescents. J Pediatr. 2007 Apr;150(4):395–399.e2. doi:10.1016/j.jpeds.2006.12.052. PMID: 17382117  Kasović M, Štefan L, Petrić V. Normative data for the 6-min walk test in 11–14 year-olds: a population-based study. BMC Pulm Med. 2021 Dec 15;21(1):297. doi:10.1186/s12890-021-01666-5. PMID: 34907238; PMCID: PMC8675802 |
| STST | 1-min STST was performed according to European Respiratory Society (ERS) standards using a chair (seat height ~46 cm) without armrests. Before the test, dyspnea (Borg scale), perceived exertion, SpO₂, heart rate (HR), and blood pressure (BP) were recorded. Participants stood up and sat down as many times as possible within 60 s, keeping arms crossed on the chest. Immediately and at 1-, 3-, and 5-minutes post-test, SpO₂, HR, BP, dyspnea, and exertion were reassessed. The total number of repetitions was used as the performance outcome. | Mobility-based resilience (functional adaptation) | Crook S, Büsching G, Schultz K, Lehbert N, Jelusic D, Keusch S, Wittmann M, Schuler M, Radtke T, Frey M, Turk A, Puhan MA, Frei A. A multicentre validation of the 1-min sit-to-stand test in patients with COPD. Eur Respir J. 2017;49(3):1601871. doi:10.1183/13993003.01871-2016.  Haile SR, Fühner T, Granacher U, Stocker J, Radtke T, Kriemler S. Reference values and validation of the 1-minute sit-to-stand test in healthy 5–16-year-old youth: a cross-sectional study. BMJ Open. 2021;11(5):e049143. doi:10.1136/bmjopen-2021-049143.  Haile SR, Fühner T, Granacher U, Stocker J, Radtke T, Kriemler S. Reference values and validation of the 1-minute sit-to-stand test in healthy 5–16-year-old youth: a cross-sectional study. BMJ Open. 2021 May 7;11(5):e049143. doi:10.1136/bmjopen-2021-049143. PMID: 33963059; PMCID: PMC8108674 |
| HST | Grip strength was assessed using a calibrated by a professional digital hand dynamometer following the recommendations of the American Society of Hand Therapists. Participants were seated with the elbow at 90°, forearm in a neutral position, and wrist slightly extended.  Handedness (right/left) was documented. Due to the fatigue within this cohort three times three maximal isometric contractions were performed per hand, separated by 1 minute. After a 1-hour rest, the procedure was repeated under identical conditions.  Values were recorded in kilograms (kg); the mean of the three highest values was used for analysis. Changes after 1 hour were interpreted as indicators of muscular fatigue. | Physical reserve (muscle strength-based resilience) | Roberts HC, Denison HJ, Martin HJ, Patel HP, Syddall H, Cooper C, Sayer AA. A review of the measurement of grip strength in clinical and epidemiological studies: towards a standardised approach. Age Ageing. 2011;40(4):423–9. doi:10.1093/ageing/afr051  Fess EE, Moran C. Clinical Assessment Recommendations. 3rd ed. Chicago: American Society of Hand Therapists; 1981  Rodríguez-Rodríguez F, Cristi-Montero C, Villa-González E, Solís-Urra P, Chillón P, Ortega FB. Handgrip strength values of Chilean children and adolescents according to chronological and biological age. PLoS One. 2018 Jul 26;13(7):e0201033. doi:10.1371/journal.pone.0201033. PMID: 30044818; PMCID: PMC6066274 |

All assessments were conducted during each visit to the Long COVID outpatient clinic.

1. **Supplement 6 – Intervention: Individualized Online Exercise Therapy (IOET)**

This supplement provides the detailed intervention description corresponding to the Methods section and our previously published protocol (B&G Bewegungstherapie und Gesundheitssport 2025; 41(01): 30–37; DOI: 10.1055/a-2479-2946).

**6.1 Session structure**

Each training session followed a structured sequence consisting of:

- **Warm-up** (joint mobility and cardiovascular activation)
- **Aerobic component** (moderate interval-based endurance)
- **Resistance/strength training** (bodyweight exercises and/or elastic resistance bands)
- **Cool-down and optional relaxation techniques**

**6.2 Intensity prescription and progression**

Session duration increased progressively from 20 to 30 minutes, with 1–3 sets of 10–15 repetitions per exercise when feasible. Training intensity was individually titrated using perceived exertion scales (Borg or PCERT), aiming for:

- **Light intensity:** Borg 6–10 (initial phases, symptom fluctuation)
- **Moderate intensity:** Borg 11–13 (primary target range)
- **Higher intensity:** Borg ≥14 (only when clinically appropriate and tolerated)

**Progression Principles and Adaptation Protocol**

Progression followed the FITT criteria (Frequency, Intensity, Type, Time), adapted continuously to symptom severity, comorbidities, and daily functional capacity. To prevent symptom exacerbation, IOET incorporated:

- A sequenced medical clearance and baseline assessment
- Continuous standardised symptom monitoring including fatigue, pain, exercise intolerance, sleep disturbances, and cognitive complaints
- Clear criteria for dose reduction or pausing sessions, e.g., symptom deterioration lasting >72h

Examples of individually applied modifications included:

- Reduced session length during symptomatic flares
- Transition from standing to seated/bed-based exercises
- Coordination tasks with lower cognitive load on high-fatigue days
- Temporary substitution of endurance modules by relaxation techniques

**6.3 Therapist training and supervision**

The intervention was delivered by experienced exercise therapists trained in pediatric Long COVID, supervised within a multidisciplinary medical team (pediatric infectious diseases, cardiology, pneumology, neurology as well as psychology). Training took place exclusively online via secure livestream.

Therapists received structured instruction covering:

- Long COVID symptom patterns and energy management
- Safe exercise prescription for post-viral conditions
- Recognition of exercise intolerance and red-flag symptoms
- Use of the modular exercise framework based on Physical Activity–Related Health Competence (PAHCO)
- As well as subjective preferences

**6.4 Modular Exercise Program**

Consistent with Bergelt et al. (2025), IOET was based on a six-module therapeutic system, with session-specific tailored module combinations:

| **Module** | **Focus Area** |
| --- | --- |
| 1 | Warm-up & mobility |
| 2 | Strength training (bodyweight / Theraband) |
| 3 | Endurance / interval-based aerobic activity |
| 4 | Balance, coordination, hand–foot motor control |
| 5 | Flexibility & mobility (cool-down) |
| 6 | Relaxation & autonomic regulation techniques |

Each participant received an individualised training plan aligned with symptoms, functional status, and personal goals.

**6.5 Individualisation and handling of symptom fluctuation**

To account for fluctuating disease trajectories, IOET used:

- Daily symptom diaries
- Interview before and after session
- Remote monitoring (heart rate, step count, SpO₂ where applicable)
- Adaptive, shared decision-making regarding session content

This approach supported self-efficacy, pacing skills, and gradual reintegration into school, physical play, and community sports.

1. **Supplement 7 – Additional Results (Quality of Life, Fatigue, Functional Outcomes and Mixed Model Results)**

This supplement presents the **detailed numerical results** for:

- PedsQL™ domain scores (parent and patient)
- Treatment effect ratings (GEEE)
- Primary and secondary functional tests (6MWT, STST, HST)
- Selected physiological markers (e.g., CK, VC)
- Linear mixed-effects model results

**7.1 Supplement Table S1: Impact of exercise therapy on quality of life and overall treatment effect, as well as physical testing**

|  | | |  | |  | | **Group 1 - 12 weeks intervention group** | | | | | | |  |  | | | **Group 2 - 6 weeks intervention group** | | | | | | |  |
| --- | --- | --- | --- | --- | --- | --- | --- | --- | --- | --- | --- | --- | --- | --- | --- | --- | --- | --- | --- | --- | --- | --- | --- | --- | --- |
|  | | |  | |  | | **1** | **2** | **3** | **4** | **5** | **6** | **7** | **Mean (CI)** | **1** | **2** | **3** | | **4** | **5** | **6** | **7** | | **Mean (CI)** |  |
|  | | **quality of life (score)*** | | | | | | | | | | | | | | | | | | | |  |  | |  |
| Problems with health and activities  (5 items) | | | Visit I | | parent | | 40 | 30 | 35 | 85 | 60 | 30 | 70 | 50 (31.3-68.7) | 55 | 25 | 35 | | 45 | 100 | 35 | 70 | 52.1 (28.3–76.0) | |  |
|  |  |  |  | | patient | | 40 | 30 | 25 | 55 | 60 | 25 | 75 | 44.3 (27.1-61.5) | 50 | 95 | 65 | | 38 | 100 | 21 | 54 | 60.4 (33.8–87.1) | |  |
|  |  |  | Visit II | | parents | | **70** | **55** | **45** | **100** | **80** | **40** | **60** | **64.3 (45.5-83.1)** | 60 | 90 | 45 | | 50 | 85 | 35 | - | 60.8 (37.5–84.2) | |  |
|  |  |  |  | | patients | | **75** | **50** | **45** | **90** | **95** | **50** | **85** | **70.0 (51.7-88.3)** | 63 | 100 | 42 | | 56 | 54 | 17 | - | 55.3 (26.8–83.9) | |  |
|  |  |  | Visit III | | parents | | **80** | **35** | **65** | **100** | **90** | **65** | **80** | **73.6 (55.3-91.9)** | **85** | **100** | **100** | | **50** | **85** | **60** | **90** | **81.4 (63.6–99.3)** | |  |
|  |  |  |  | | patient | | **75** | **60** | **70** | **100** | **90** | **60** | **100** | **79.3 (61.3-97.3)** | **100** | **100** | **100** | | **58** | **79** | **50** | **100** | **83.9 (63.6–104.1)** | |  |
|  |  |  | Visit IV | | parent | | **75** | **50** | **60** | **85** | **100** | **75** | **-** | **74.2 (55.8-92.3)** | **50** | **100** | **85** | | **30** | **100** | **55** | **100** | **74.3 (47.5–101.0)** | |  |
|  |  |  |  | | patient | | **75** | **40** | **35** | **90** | **95** | **60** | **-** | **65.8 (41.2-90.5)** | **42** | **100** | **71** | | **25** | **75** | **42** | **100** | **65.0 (37.7–92.3)** | |  |
| problems with mood and feelings  (4 items) | | | Visit I | | parent | | 69 | 50 | 44 | 88 | 44 | 25 | 62 | 54.6 (35.2-74.0) | 100 | 38 | 75 | | 19 | 81 | 44 | 44 | 57.3 (30.9–83.7) | |  |
|  |  |  |  | | patient | | 56 | 50 | 50 | 69 | 25 | 25 | 69 | 49.1 (33.8-64.5) | 50 | 31 | 63 | | 38 | 63 | 19 | 75 | 48.4 (29.9–67.0) | |  |
|  |  |  | Visit II | | parents | | **50** | **50** | **56** | **100** | **81** | **75** | **94** | **72.3 (55.1-89.5)** | 94 | 50 | 94 | | 25 | 75 | 44 | - | 63.7 (33.9–93.5) | |  |
|  |  |  |  | | patients | | **69** | **88** | **56** | **75** | **38** | **75** | **81** | **68.9 (53.2-84.6)** | **94** | **38** | **94** | | **50** | **56** | **19** | **-** | **58.5 (26.7–90.3)** | |  |
|  |  |  | Visit III | | parents | | **100** | **69** | **69** | **100** | **94** | **63** | **75** | **81.4 (67.3-95.6)** | **100** | **100** | **100** | | **44** | **81** | **31** | **81** | **76.7 (50.5–103.0)** | |  |
|  |  |  |  | | patient | | **94** | **63** | **56** | **75** | **38** | **75** | **94** | **70.7** (52.0-89.5) | **100** | **100** | **100** | | **75** | **88** | **38** | **100** | **85.9 (64.5–107.3)** | |  |
|  |  |  | Visit IV | | parent | | **69** | **69** | **63** | **75** | **100** | **56** | **-** | **72.0** (56.1-87.9) | **63** | **100** | **100** | | **44** | **75** | **44** | **75** | **71.6 (50.1–93.0)** | |  |
|  |  |  |  | | patient | | **88** | **75** | **56** | **75** | **56** | **50** | **-** | **66.7** (51.1-82.2) | **75** | **100** | **100** | | **18** | **100** | **38** | **88** | **74.1 (43.3–105.0)** | |  |
| Problems with peers  (3 items) | | | Visit I | | parent | | 17 | 25 | 17 | 100 | 25 | 22 | 58 | **37.7** (9.2-66.3) | 25 | 25 | 33 | | 17 | 58 | 25 | 42 | 32.1 (19.4–45.0) | |  |
|  |  |  |  | | patient | | 42 | 8 | 17 | 100 | 17 | 17 | 58 | **37.0** (6.6-67.4) | 25 | 33 | 33 | | 8 | 50 | 8 | 58 | 30.7 (13.0–48.4) | |  |
|  |  |  | Visit II | | parents | | **42** | **42** | **50** | **100** | **42** | **42** | **33** | **50.1** (29.3-71.0) | 25 | 42 | 33 | | 17 | 67 | 0 | - | 30.7 (6.7–54.7) | |  |
|  |  |  |  | | patients | | **67** | **50** | **50** | **100** | **33** | **67** | **67** | **62.0** (42.6-81.4) | 25 | 25 | 33 | | 17 | 52 | 0 | - | 25.3 (7.3–43.4) | |  |
|  |  |  | Visit III | | parents | | **100** | **33** | **42** | **100** | **42** | **75** | **67** | **65.6** (39.9-91.3) | **100** | **100** | **100** | | **50** | **50** | **17** | **100** | **73.9 (42.0–105.7)** | |  |
|  |  |  |  | | patient | | **100** | **42** | **58** | **100** | **50** | **67** | **75** | **70.3** (49.1-91.5) | **100** | **100** | **83** | | **67** | **83** | **17** | **100** | **78.6 (51.0–106.2)** | |  |
|  |  |  | Visit IV | | parent | | **67** | **50** | **42** | **100** | **75** | **58** | **-** | **65.3** (43.7-87.0) | **42** | **100** | **100** | | **25** | **67** | **17** | **100** | **64.4 (30.4–98.4)** | |  |
|  | | |  | | patient | | **67** | **33** | **42** | **100** | **67** | **58** | **-** | **61.2** (36.6-85.8) | **42** | **100** | **100** | | **17** | **100** | **33** | **92** | **69.1 (35.1–103.2)** | |  |
| Problems with school (3 items) | | | Visit I | | parent | | 17 | 25 | 17 | 100 | 25 | 33 | 58 | **39.3** (11.3-67.2) | 25 | 25 | 33 | | 17 | 58 | 25 | 42 | 48.4 (29.9–67.0) | |  |
|  |  |  |  | | patient | | 42 | 8 | 17 | 100 | 17 | 17 | 58 | **37.0** (6.6-67.4) | 25 | 33 | 33 | | 8 | 50 | 8 | 58 | 63.7 (33.9–93.5) | |  |
|  |  |  | Visit II | | parents | | **42** | **42** | **50** | **100** | **42** | **42** | **33** | **50.1** (29.3-71.0) | 25 | 42 | 33 | | 17 | 67 | 0 | - | 58.5 (26.7–90.3) | |  |
|  |  |  |  | | patients | | **67** | **50** | **50** | **100** | **33** | **67** | **67** | **62.0** (42.6-81.4) | 25 | 25 | 33 | | 17 | 52 | 0 | - | 25.3 (7.3–43.4) | |  |
|  |  |  | Visit III | | parents | | **100** | **33** | **42** | **100** | **42** | **75** | **67** | **65.6** (39.9-91.3) | **100** | **100** | **100** | | **50** | **50** | **17** | **100** | **73.9 (42.0–105.7)** | |  |
|  |  |  |  | | patient | | **100** | **42** | **58** | **100** | **50** | **67** | **75** | **70.3** (49.1-91.5) | **100** | **100** | **83** | | **67** | **83** | **17** | **100** | **78.6 (51.0–106.2)** | |  |
|  |  |  | Visit IV | | Parent | | **67** | **50** | **42** | **100** | **75** | **58** | **-** | **65.3** (43.7-87.0) | **42** | **100** | **100** | | **25** | **67** | **17** | **100** | **64.4 (30.4–98.4)** | |  |
|  | | |  | | Patient | | **67** | **33** | **42** | **100** | **67** | **58** | **-** | **61.2** (36.6-85.8) | **42** | **100** | **100** | | **17** | **100** | **33** | **92** | **69.1 (35.1–103.2)** | |  |
| Problems with general fatigue  (6 items) | | | Visit I | | Parent | | 21 | 33 | 21 | 96 | 17 | 33 | 50 | 38.7 (13.2-64.3) | 75 | 29 | 50 | | 25 | 83 | 29 | 21 | 44.6 (21.1–68.0) | |  |
|  |  |  |  | | Patients | | 42 | 42 | 29 | 83 | 42 | 21 | 63 | 46.0 (26.7-65.3) | 83 | 46 | 42 | | 42 | 58 | 13 | 71 | 50.7 (29.7–71.6) | |  |
|  |  |  | Visit II | | parent | | **67** | **46** | **38** | **92** | **67** | **50** | **50** | **58.6** (41.7-75.4) | 79 | 67 | 42 | | 25 | 67 | 21 | - | 50.1 (24.7–75.7) | |  |
|  |  |  |  | | patient | | **58** | **54** | **42** | **92** | **46** | **63** | **71** | **60.9** (45.3-76.5) | 79 | 38 | 38 | | 21 | 60 | 13 | - | 41.5 (15.8–67.2) | |  |
|  |  |  | Visit III | | parents | | **83** | **46** | **58** | **96** | **50** | **67** | **63** | **66.1** (49.6-82.7) | **88** | **96** | **67** | | **50** | **96** | **25** | **83** | **72.1 (47.5–96.8)** | |  |
|  |  |  |  | | patients | | **83** | **67** | **46** | **100** | **54** | **71** | **75** | **70.9** (54.3-87.5) | **88** | **96** | **83** | | **50** | **91** | **25** | **100** | **76.1 (50.3–102.0)** | |  |
|  |  |  | Visit IV | | parent | | **67** | **50** | **50** | **92** | **67** | **50** | **-** | **62.7** (45.2-80.1) | **46** | **100** | **71** | | **25** | **83** | **25** | **83** | **61.9 (34.1–89.6)** | |  |
|  | | |  | | Patients | | **71** | **50** | **25** | **88** | **79** | **42** | **-** | **59.2** (33.9-84.5) | **54** | **100** | **67** | | **25** | **79** | **42** | **100** | **66.7 (40.3–93.1)** | |  |
| Problems with fatigue / need to rest  (6 items) | | | Visit I | | Parent | | 42 | 58 | 25 | 92 | 46 | 38 | 33 | 47.7 (27.3-68.2) | 67 | 33 | 63 | | 50 | 38 | 25 | 25 | 43.0 (27.0–59.0) | |  |
|  |  |  |  | | Patients | | 29 | 25 | 33 | 75 | 71 | 29 | 42 | 43.4 (24.1-62.8) | 58 | 29 | 71 | | 54 | 42 | 29 | 38 | 45.9 (31.3–60.4) | |  |
|  |  |  | Visit II | | parent | | **46** | **54** | **38** | **92** | **83** | **67** | **46** | **60.9** (42.0-79.8) | 58 | 33 | 75 | | 46 | 50 | 33 | - | 49.2 (32.4–66.0) | |  |
|  |  |  |  | | patient | | **71** | **42** | **21** | **88** | **79** | **33** | **79** | **59.0** (34.6-83.5) | 58 | 33 | 71 | | 38 | 38 | 21 | - | 43.2 (24.1–62.2) | |  |
|  |  |  | Visit III | | parents | | **75** | **33** | **54** | **83** | **83** | **75** | **63** | **66.6** (49.8-83.4) | **79** | **67** | **92** | | **50** | **50** | **38** | **75** | **25.3 (7.3–43.4)** | |  |
|  |  |  |  | | patients | | **75** | **50** | **54** | **96** | **79** | **50** | **88** | **70.3** (52.7-87.8) | **79** | **67** | **92** | | **67** | **63** | **67** | **71** | **73.9 (42.0–105.7)** | |  |
|  |  |  | Visit IV | | parent | | **58** | **50** | **38** | **71** | **75** | **54** | **-** | **57.7** (43.3-72.0) | **54** | **100** | **79** | | **46** | **38** | **42** | **88** | **78.6 (51.0–106.2)** | |  |
|  | | |  | | Patients | | **54** | **33** | **25** | **83** | **92** | **25** | **-** | **52.0** (20.9-83.1) | **58** | **100** | **67** | | **29** | **38** | **67** | **83** | **64.4 (30.4–98.4)** | |  |
| Problems with mental fatigue  (6 items) | | | Visit I | | Parent | | 42 | 50 | 21 | 100 | 17 | 50 | 54 | 47.7 (22.5-73.0) | 38 | 33 | 67 | | 38 | 88 | 13 | 54 | 69.1 (35.1–103.2) | |  |
|  |  |  |  | | Patients | | 33 | 38 | 38 | 100 | 54 | 33 | 63 | 51.3 (28.8-73.8) | 46 | 29 | 50 | | 50 | 83 | 0 | 50 | 44.6 (21.1–68.0) | |  |
|  |  |  | Visit II | | parent | | **46** | **79** | **50** | **100** | **50** | **63** | **63** | **64.4** (46.6-82.3) | 63 | 75 | 63 | | 50 | 63 | 33 | - | 50.7 (29.7–71.8) | |  |
|  |  |  |  | | patient | | **75** | **38** | **38** | **100** | **54** | **42** | **75** | **60.3** (38.3-82.2) | 79 | 38 | 71 | | 67 | 88 | 0 | - | 50.2 (24.7–75.7) | |  |
|  |  |  | Visit III | | parents | | **67** | **46** | **63** | **100** | **63** | **67** | **75** | **68.7** (53.6-83.8) | **100** | **79** | **75** | | **54** | **96** | **29** | **100** | **41.5 (15.8–67.2)** | |  |
|  |  |  |  | | patients | | **63** | **88** | **71** | **100** | **75** | **50** | **75** | **74.6** (59.6-89.6) | **100** | **92** | **88** | | **100** | **100** | **33** | **96** | **72.1 (47.5–96.8)** | |  |
|  |  |  | Visit IV | | parent | | **67** | **63** | **50** | **100** | **96** | **63** | **-** | **73.2** (52.1-94.3) | **96** | **100** | **96** | | **50** | **96** | **29** | **100** | **76.1 (50.3–102.0)** | |  |
|  |  |  |  | | Patients | | **71** | **33** | **38** | **100** | **96** | **42** | **-** | **63.3** (31.9-94.8) | **63** | **100** | **92** | | **50** | **83** | **17** | **100** | **61.9 (34.1–89.6)** | |  |
|  | |  | | | **Physical testing** | | | | | | | | | | | | | | | | | | | | |
| 6-Minute-Walking-Test | | | | | | |  |  |  |  |  |  |  |  |  |  |  |  | |  |  |  |  |  | |
|  | | | | Visit I | | | | 500 | 242 | 404 | 357 | 599 | 334 | 336 | 396.0 (286.1–505.9) | 392 | 635 | 397 | | 482 | 432 | 285 | 380 | 429.0 (328.6–529.5) | |
|  | | | | Visit II | | | | **714** | **200** | **481** | **443** | **730** | **745** | **534** | **549.6 (366.0–733.1)** | 392 | 587 | 383 | | 586 | 570 | 386 | 480 | 483.4 (393.5–573.3) | |
|  | | | | Visit III | | | | **894** | **423** | **585** | **587** | **763** | **369** | **878** | **642.7 (449.2–836.2)** | **922** | **844** | **459** | | **867** | **653** | **534** | **997** | **753.7 (563.5–943.9)** | |
|  | | | | Visit IV | | | | **632** | **216** | **383** | **979** | **661** | **623** | **820** | **616.3 (380.5–852.1)** | **401** | **800** | **765** | | **516** | **570** | **500** | **659** | **601.6 (466.4–736.8)** | |
| Sit-to-Stand | | | |  | | |  |  |  |  |  |  |  |  |  |  |  |  | |  |  |  |  |  | |
|  | | | | Visit I | | | | 21 | 22 | 15 | 50 | 27 | 21 | 22 | 25.43 (14.9–35.96) | 17 | 34 | 30 | | 25 | 15 | 9 | 21 | 21.6 (13.5–29.7) | |
|  | | | | Visit II | | | | **24** | **24** | **20** | **63** | **33** | **33** | **34** | **33.0 (19.8–46.3)** | 17 | 37 | 34 | | 24 | 17 | 11 | 20 | 22.9 (14.1–31.6) | |
|  | | | | Visit III | | | | **32** | **28** | **20** | **116** | **28** | **31** | **34** | **41.3 (10.5–72.0)** | **23** | **48** | **44** | | **34** | **32** | **32** | **49** | **37.4 (28.4–46.4)** | |
|  | | | | Visit IV | | | | **24** | **24** | **14** | **76** | **34** | **22** | **34** | **32.6 (13.7–51.4)** | **21** | **43** | **33** | | **-** | **30** | **30** | **33** | **31.7 (24.2–39.1)** | |
| Hand-Grip | | | |  | | |  |  |  |  |  |  |  |  |  |  |  |  | |  |  |  |  |  | |
|  | | | | Visit I | | | | 13 | 8 | 15 | 22 | 31 | 11 | 16 | 16.6 (9.4–23.7) | 15 | 29 | 18 | | 18 | 22 | 10 | 9 | 17.3 (10.9–23.7) | |
|  | | | | Visit II | | | | **31** | **13** | **31** | **22** | **37** | **17** | **26** | **25.3 (17.4–33.2)** | 15 | 30 | 16 | | 23 | 20 | 10 | 9 | 17.6 (10.7–24.4) | |
|  | | | | Visit III | | | | **43** | **16** | **31** | **24** | **32** | **21** | **29** | **28.0 (19.9–36.1)** | **20** | **36** | **19** | | **28** | **33** | **18** | **12** | **23.7 (15.6–31.8)** | |
|  | | | | Visit IV | | | | **38** | **12** | **28** | **34** | **30** | **20** | **28** | **27.1 (19.1–35.2)** | **19** | **34** | **19** | | **23** | **28** | **17** | **15** | **22.1 (15.9–28.4)** | |
| VC (%) | | | |  | | |  |  |  |  |  |  |  |  |  |  |  |  | |  |  |  |  |  | |
|  | | | | Visit I | | | | 4,26 | 2,66 | 2,24 | 2,98 | 2,24 | 2,78 | 3,67 | 3.0 (2.3–3.7) | 3,04 | 2,72 | 3,94 | | 2,81 | 2,39 | 2,61 | 1,65 | 2.7 (2.1–3.4) | |
|  | | | | Visit II | | | | **4,3** | **3,29** | **3,89** | **2,98** | **3,2** | **2,29** | **3,54** | **3.4 (2.8–4.0)** | 3,54 | 3,44 | 3,33 | | 2,93 | 2,16 | 2,83 | - | 3.0 (2.5–3.6) | |
|  | | | | Visit III | | | | **4,42** | **3,29** | **3,89** | **2,98** | - | **2,39** | **3,44** | **3.4 (2.7–4.1)** | **3,44** | - | **3,26** | | **2,74** | **2,16** | **2,83** | **0,61** | **2.5 (1.4–3.6)** | |
|  | | | | Visit IV | | | | **4,95** | **3,75** | **4,17** | **2,44** | - | **2,73** | **3,67** | **3.6 (2.7–4.6)** | **3,44** | **4,78** | **2,88** | | **2,93** | - | - | **1,61** | **3.1 (1.7–4.6)** | |
| CK (U/l) | | | |  | | |  |  |  |  |  |  |  |  |  |  |  |  | |  |  |  |  |  | |
|  | | | | Visit I | | |  | 186 | 101 | 136 | 119 | 89 | 146 | 112 | 127.0 (97.0–157.0) | 129 | 80 | 93 | | 66 | 49 | 108 | 108 | 90.4 (65.0–115.9) | |
|  | | | | Visit II | | |  | **214** | **132** | **133** | **284** | **103** | **222** | **152** | **177.1 (117.6–236.7)** | 129 | 80 | 78 | | 70 | 68 | 108 | 129 | 94.6 (69.7–119.5) | |
|  | | | | Visit III | | |  | **175** | **198** | **160** | **107** | **92** | **114** | **111** | **136.7 (99.3–174.1)** | **169** | **75** | **105** | | **77** | **75** | **150** | **177** | **118.3 (75.8–160.8)** | |
|  | | | | Visit IV | | |  | **169** | **120** | **214** | **182** | **85** | **121** | **80** | **138.7 (91.8–185.7)** | **124** | **75** | **93** | | **55** | **60** | **43** | **101** | **78.7 (52.1–105.3)** | |
|  | |  | | | * mean value of visits from PedsQL (higher scores (100 the highest) indicate better HRQoL / health related quality of life); all values are rounded to a whole number, CI rounded to one numer after the point; bold vales are taken after exercise therapy | | | | | | | | | | | | | | | | | | | | |

**7.2 Supplement Table S 2 Groups characteristics, impact of exercise therapy on participation in daily life and treatment effect**

|  | |  | |  | **Group 1 - 12 weeks intervention group** | | | | | | | | | | | **Group 2 - 6 weeks intervention group** | | | | | | | | | | |  |
| --- | --- | --- | --- | --- | --- | --- | --- | --- | --- | --- | --- | --- | --- | --- | --- | --- | --- | --- | --- | --- | --- | --- | --- | --- | --- | --- | --- |
|  | |  | |  | **1** | **2** | | **3** | **4** | | **5** | | **6** | | **7** | **1** | **2** | **3** | **4** | | **5** | | **6** | | **7** | |  |
| **Treatment effect*** | | | | | | | | | | | | | | | | | | | | | | | | | | |  |
| Improve-ment* | | Visit II | | parent | | **4** | **4** | **3** | | **0** | **5** | | **5** | | **6** | | no values as there had been no intervention so far. | | | | | | | | | | |
|  |  |  | | patient | | **6** | **2** | **6** | | **1** | **3** | | **7** | | **6** | |  |  |  |  |  |  |  |  |  |  |  |
|  |  | Visit III | | parent | | **8** | **6** | **5** | | **4** | **5** | | **7** | | **5** | | **8** | **8** | **5** | | **8** | | **0** | | **7** | | **7** |
|  |  |  | | patient | | **8** | **8** | **7** | | **8** | **7** | | **9** | | **9** | | **9** | **10** | **8** | | **6** | | **5** | | **8** | | **10** |
|  |  | Visit IV | | parent | | **8** | **6** | **6** | | **1** | **10** | | **7** | | **10** | | **6** | **10** | **8** | | **2** | | **1** | | **10** | | **9** |
|  |  |  | | patient | | **8** | **5** | **8** | | **8** | **10** | | **7** | | **10** | | **9** | **10** | **4** | | **2** | | **2** | | **10** | | **9** |
| Deterio-ration * | | Visit II | | parent | | **0** | **0** | **0** | | **0** | **0** | | **0** | | **2** | | no values as there had been no intervention so far. | | | | | | | | | | |
|  |  |  | | patient | | **1** | **0** | **2** | | **0** | **0** | | **3** | | **0** | |  |  |  |  |  |  |  |  |  |  |  |
|  |  | Visit III | | parent | | **0** | **0** | **0** | | **0** | **0** | | **1** | | **1** | | **0** | **0** | **0** | | **2** | | **0** | | **0** | | **1** |
|  |  |  | | patient | | **0** | **0** | **2** | | **0** | **0** | | **2** | | **1** | | **0** | **0** | **0** | | **4** | | **0** | | **0** | | **1** |
|  |  | Visit IV | | parent | | **0** | **0** | **6** | | **0** | **0** | | **0** | | **0** | | **0** | **0** | **3** | | **0** | | **0** | | **0** | | **0** |
|  |  |  | | patient | | **0** | **0** | **0** | | **5** | **0** | | **4** | | **0** | | **0** | **0** | **0** | | **1** | | **0** | | **0** | | **0** |
| * all values are rounded to a whole number, bold values are taken after exercise therapy – one being the lowest and 10 the highest score for improvement or deterioration  **7.3 Supplement Table S 3 Detailed Results of the Linear Mixed Effects Model**  **A)** | | | | | | | | | | | | | | | | | | | | | | | | | | | |

|  | **6MWT** | | | | **Handgrip** | | | | **Sit-to-Stand** | | | | **CK** | | | |
| --- | --- | --- | --- | --- | --- | --- | --- | --- | --- | --- | --- | --- | --- | --- | --- | --- |
| *Predictors* | *Estimates* | *std. Error* | *CI* | *p* | *Estimates* | *std. Error* | *CI* | *p* | *Estimates* | *std. Error* | *CI* | *p* | *Estimates* | *std. Error* | *CI* | *p* |
| Intercept | 396.13 | 66.44 | 262.39 – 529.87 | <0.001 | 16.41 | 3.00 | 10.38 – 22.45 | <0.001 | 25.43 | 6.23 | 12.89 – 37.97 | <0.001 | 127.00 | 15.72 | 95.36 – 158.64 | <0.001 |
| Visit 2 | 153.37 | 76.05 | 0.29 – 306.45 | 0.050 | 8.73 | 1.89 | 4.93 – 12.53 | <0.001 | 7.57 | 4.33 | -1.14 – 16.29 | 0.087 | 50.14 | 16.95 | 16.02 – 84.27 | 0.005 |
| Visit 3 | 246.66 | 76.05 | 93.58 – 399.74 | 0.002 | 11.41 | 1.89 | 7.62 – 15.21 | <0.001 | 15.86 | 4.33 | 7.14 – 24.57 | 0.001 | 9.71 | 16.95 | -24.41 – 43.84 | 0.569 |
| Visit 4 | 220.13 | 76.05 | 67.05 – 373.21 | 0.006 | 9.27 | 1.89 | 5.47 – 13.07 | <0.001 | 7.14 | 4.33 | -1.57 – 15.86 | 0.106 | 11.71 | 16.95 | -22.41 – 45.84 | 0.493 |
| Group (Reference: 6 Weeks) | 32.87 | 93.96 | -156.26 – 222.00 | 0.728 | 0.94 | 4.24 | -7.59 – 9.48 | 0.825 | -3.86 | 8.81 | -21.59 – 13.87 | 0.664 | -36.57 | 22.23 | -81.31 – 8.17 | 0.107 |
| Visit 2 * Group (Reference: 6 Weeks) | -99.10 | 107.55 | -315.59 – 117.39 | 0.362 | -8.51 | 2.67 | -13.89 – -3.14 | 0.003 | -6.29 | 6.12 | -18.61 – 6.04 | 0.310 | -46.00 | 23.98 | -94.26 – 2.26 | 0.061 |
| Visit 3 * Group (Reference: 6 Weeks) | 78.06 | 107.55 | -138.43 – 294.54 | 0.472 | -5.09 | 2.67 | -10.46 – 0.29 | 0.063 | 0.00 | 6.12 | -12.32 – 12.32 | 1.000 | 18.14 | 23.98 | -30.12 – 66.40 | 0.453 |
| Visit 4 * Group (Reference: 6 Weeks) | -47.66 | 107.55 | -264.14 – 168.83 | 0.660 | -4.61 | 2.67 | -9.99 – 0.76 | 0.090 | 1.61 | 6.12 | -10.72 – 13.93 | 0.794 | -23.43 | 23.98 | -71.69 – 24.83 | 0.334 |
| **Random Effects** | | | | | | | | | | | | | | | | |
| σ^2^ | 20241.92 | | | | 12.46 | | | | 65.61 | | | | 1006.00 | | | |
| τ_00_ | 10657.85 _ID_ | | | | 50.49 _ID_ | | | | 205.95 _ID_ | | | | 723.25 _ID_ | | | |
| ICC | 0.34 | | | | 0.80 | | | | 0.76 | | | | 0.42 | | | |
| N | 14 _ID_ | | | | 14 _ID_ | | | | 14 _ID_ | | | | 14 _ID_ | | | |
| Observations | 56 | | | | 56 | | | | 56 | | | | 56 | | | |
| Marginal R^2^ / Conditional R^2^ | 0.290 / 0.535 | | | | 0.211 / 0.844 | | | | 0.137 / 0.792 | | | | 0.345 / 0.619 | | | |

σ2 = residual (within-group) variance, τ00 = random intercept (between-group) variance, Intraclass Correllation Coefficient (between Group difference), N = number of patients,; arginal R2 = variance explained by fixed effects only, Conditional R2 = variance explained by fixed effects only

**B)**

|  | **QoL Patient** | | | | **QoL Parent** | | | | **Fatigue Patient** | | | | **Fatigue Parent** | | | |
| --- | --- | --- | --- | --- | --- | --- | --- | --- | --- | --- | --- | --- | --- | --- | --- | --- |
| *Predictors* | *Estimates* | *std. Error* | *CI* | *p* | *Estimates* | *std. Error* | *CI* | *p* | *Estimates* | *std. Error* | *CI* | *p* | *Estimates* | *std. Error* | *CI* | *p* |
| Intercept | 54.00 | 7.32 | 39.26 – 68.74 | <0.001 | 56.43 | 7.64 | 41.05 – 71.81 | <0.001 | 48.14 | 7.71 | 32.62 – 63.66 | <0.001 | 44.71 | 7.35 | 29.92 – 59.51 | <0.001 |
| Visit 2 | 19.86 | 6.40 | 6.97 – 32.74 | 0.003 | 11.43 | 6.70 | -2.06 – 24.91 | 0.095 | 11.86 | 6.17 | -0.56 – 24.27 | 0.061 | 16.71 | 6.02 | 4.60 – 28.82 | 0.008 |
| Visit 3 | 25.57 | 6.40 | 12.69 – 38.46 | <0.001 | 22.14 | 6.70 | 8.66 – 35.63 | 0.002 | 23.71 | 6.17 | 11.30 – 36.13 | <0.001 | 21.00 | 6.02 | 8.89 – 33.11 | 0.001 |
| Visit 4 | 14.72 | 6.40 | 1.83 – 27.60 | 0.026 | 14.09 | 6.70 | 0.61 – 27.58 | 0.041 | 13.47 | 6.17 | 1.05 – 25.88 | 0.034 | 19.79 | 6.02 | 7.68 – 31.90 | 0.002 |
| Group (Reference: 6 Weeks) | -1.14 | 10.35 | -21.98 – 19.70 | 0.913 | -3.14 | 10.81 | -24.90 – 18.61 | 0.772 | -1.29 | 10.90 | -23.24 – 20.66 | 0.907 | 0.29 | 10.39 | -20.63 – 21.21 | 0.978 |
| Visit 2 * Group (Reference: 6 Weeks) | -25.56 | 9.05 | -43.78 – -7.34 | 0.007 | -7.72 | 9.47 | -26.79 – 11.35 | 0.420 | -11.47 | 8.72 | -29.02 – 6.09 | 0.195 | -10.07 | 8.51 | -27.20 – 7.06 | 0.243 |
| Visit 3 * Group (Reference: 6 Weeks) | 5.57 | 9.05 | -12.65 – 23.79 | 0.541 | 3.14 | 9.47 | -15.93 – 22.21 | 0.742 | 7.71 | 8.72 | -9.84 – 25.27 | 0.381 | 5.00 | 8.51 | -12.13 – 22.13 | 0.560 |
| Visit 4 * Group (Reference: 6 Weeks) | 5.85 | 9.05 | -12.37 – 24.07 | 0.521 | 8.48 | 9.47 | -10.59 – 27.55 | 0.375 | 6.82 | 8.72 | -10.74 – 24.37 | 0.438 | 5.50 | 8.51 | -11.63 – 22.63 | 0.521 |
| **Random Effects** | | | | | | | | | | | | | | | | |
| σ^2^ | 143.39 | | | | 157.06 | | | | 133.14 | | | | 126.69 | | | |
| τ_00_ | 231.80 _ID_ | | | | 251.68 _ID_ | | | | 283.04 _ID_ | | | | 251.37 _ID_ | | | |
| ICC | 0.62 | | | | 0.62 | | | | 0.68 | | | | 0.66 | | | |
| N | 14 _ID_ | | | | 14 _ID_ | | | | 14 _ID_ | | | | 14 _ID_ | | | |
| Observations | 56 | | | | 56 | | | | 56 | | | | 56 | | | |
| Marginal R^2^ / Conditional R^2^ | 0.306 / 0.735 | | | | 0.191 / 0.689 | | | | 0.235 / 0.755 | | | | 0.213 / 0.736 | | | |

σ^2^ = residual (within-group) variance, τ_00_  = random intercept (between-group) variance, Intraclass Correllation Coefficient (between Group difference), N = number of patients,; Marginal R^2^  = variance explained by fixed effects only, Conditional R^2^ = variance explained by fixed effects only
